# The L-shaped correlation between systolic blood pressure and short-term and long-term mortality in patients with cerebral hemorrhage

**DOI:** 10.1186/s12883-023-03271-x

**Published:** 2023-06-14

**Authors:** Jiang Xu, Zhiping Xie, Kang Chen, Shihai Lan, Gang Liao, Shan Xu, Xuanyong Yang, Hai Luo

**Affiliations:** 1 Department of Neurosurgery, The First Affiliated Hospital, Nanchang University, Nanchang, Jiangxi 330006 People’s Republic of China; 2 Department of Neurosurgery, Jiangxi Provincial People’s Hospital, The First Affiliated Hospital of Nanchang Medical College, Nanchang, 330006 China; 3 Institute of Medicine, Nanchang University, Nanchang, Jiangxi 330006 People’s Republic of China; 4 Department of Pathology, The First Affiliated Hospital, Nanchang University, Nanchang, Jiangxi 330006 People’s Republic of China

**Keywords:** Cerebral hemorrhage, Blood pressure, Intensive care unit, Prognosis, Mortality

## Abstract

**Background:**

A large amount of evidence has shown the necessity of lowering blood pressure (BP) in patients with acute cerebral hemorrhage, but whether reducing BP contributes to lower short-term and long-term mortality in these patients remains uncertain.

**Aims:**

We aimed to explore the association between BP, including systolic and diastolic BP, during intensive care unit (ICU) admission and 1-month and 1-year mortality after discharge of patients with cerebral hemorrhage.

**Methods:**

A total of 1085 patients with cerebral hemorrhage were obtained from the Medical Information Mart for Intensive Care III (MIMIC-III) database. Maximum and minimum values of systolic and diastolic BP in these patients during their ICU stay were recorded, and endpoint events were defined as the 1-month mortality and 1-year mortality after the first admission. Multivariable adjusted models were performed for the association of BP with the endpoint events.

**Results:**

We observed that patients with hypertension were likely to be older, Asian or Black and had worse health insurance and higher systolic BP than those without hypertension. The logistic regression analysis showed inverse relationships between systolic BP-min (odds ratio (OR) = 0.986, 95% CI 0.983–0.989, *P* < 0.001) and diastolic BP-min (OR = 0.975, 95% CI 0.968–0.981, *P* < 0.001) and risks of 1-month, as well as 1-year mortality when controlling for confounders including age, sex, race, insurance, heart failure, myocardial infarct, malignancy, cerebral infarction, diabetes and chronic kidney disease. Furthermore, smooth curve analysis suggested an approximate L-shaped association of systolic BP with the risk of 1-month mortality and 1-year mortality. Reducing systolic BP in the range of 100–150 mmHg has a lower death risk in these patients with cerebral hemorrhage.

**Conclusion:**

We observed an L-shaped association between systolic BP levels and the risks of 1-month and 1-year mortality in patients with cerebral hemorrhage, which supported that lowering BP when treating an acute hypertensive response could reduce short-term and long-term mortality.

## Introduction

Because of the heterogeneity in stroke causes and comorbidities, blood pressure BP management is complex and controversial [1, 2]. Although previous research guidelines for the early management of patients with acute ischemic stroke recommended a BP goal of less than 180/105 mmHg in stroke patients who underwent successful reperfusion [3], lower BP goals have been proposed after considering reperfusion injury and hemorrhagic complications. For instance, the DAWN study recommended that the systolic BP goal should be less than 140 mmHg after successful revascularization of patients with stroke [4]. Importantly, long-term high BP can cause vitreous degeneration of cerebral artery, decreased elasticity and increased fragility of blood vessel wall [5]. When the blood pressure rises suddenly, the fragile blood vessels are easy to break and bleed. Thus active control of BP is of great significance to the prevention and treatment of cerebral hemorrhage.

Hypertension often suggests a poor prognosis in patients with spontaneous intracerebral hemorrhage (ICH) [6–9], but the current studies on lowering BP for ICH patients are conflicting. For example, some clinical investigations have suggested that systolic BP reduction resulted in clinical improvement of ICH patients [10–12] and target SBP level of 130–139 mm Hg is likely to provide maximum benefit in acute ICH [13]. One observational study also reported that inadequate BP control during follow-up were associated with higher risk of both lobar and nonlobar ICH recurrence [14]. Another sutdy suggested that an increased mean BP on admission in putaminal and thalamic hemorrhage were related to increased mortality [15]. While others reported that reducing systolic BP had no benefits or had adverse effects on the clinical outcomes of ICH patients [16–18]. A previous meta-analysis including 32 trials involving 9008 patients suggested that BP showed a U- or J-shaped relationship with early death in acute ischemic or hemorrhagic stroke. They concluded that sharp increases or declines in BP are related to worse outcomes, and modest reductions may reduce the death risk [19].

To further investigate the relationships between BP and short-term and long-term prognosis, we included a total of 1085 patients with cerebral hemorrhage from the Medical Information Mart for Intensive Care III (MIMIC-III) database, which provided detailed clinical variables, including BP, comorbidities and blood biochemical analysis. Our study purpose was to determine the associations between maximum and minimum values of BP during intensive care unit (ICU) admission and poor outcomes (1-month mortality and 1-year mortality) after discharge of patients with cerebral hemorrhage.

## Materials and methods

### Study samples

**Fig. 1:**
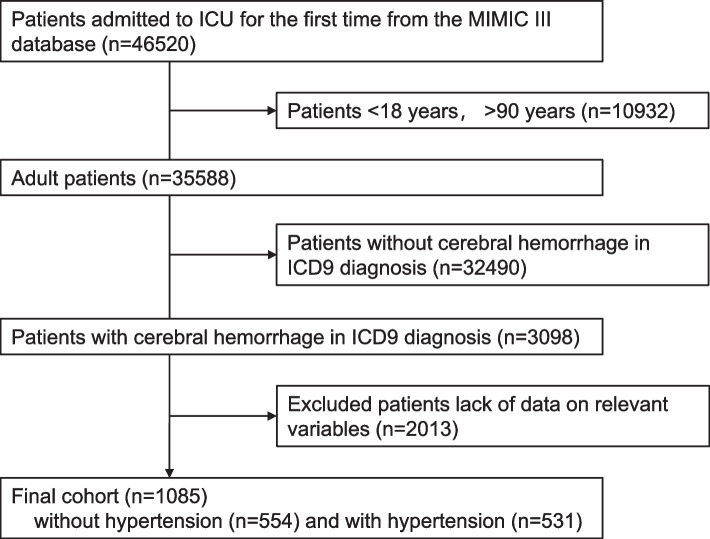
Study flow diagram of the present study

We performed a retrospective analysis on MIMIC-III (v1.4) [20]. The database comprehensively included high-quality data with various clinical variables from ICU patients who were admitted to various ICUs in Boston, Massachusetts, between 2001 and 2012. Data can be extracted and analyzed for free by each user only after passing the course “Protecting Human Research Participants” on the website of the National Institutes of Health (NIH). Our study is consistent with the Reporting of Studies Conducted using Observational Routinely Collected Health Data (RECORD) statement [21]. Our study only included adult patients who were diagnosed with cerebral hemorrhage. The inclusion and exclusion criteria were as follows: 1) if the patient had been hospitalized several times, only the first admission information was analyzed, and the remaining hospitalization information was excluded; 2) patients aged ≥ 18 years and ≤ 90 years were included. We excluded adult patients aged > 90 years because the MIMIC-III database has changed these patient's dates of birth to anonymize their age. 3) Patients who were diagnosed with cerebral hemorrhage according to the International Classification of Diseases 9th Edition (ICD-9) code were enrolled in our analysis. 4) Because the MIMIC-III database has missing data, we excluded patients with missing data for relevant variables. Finally, a total of 1085 ICU patients with cerebral hemorrhage were analyzed in our cohort study, as shown in Fig. 1. According to the Declaration of Helsinki, all methods were carried out in accordance with the guideline and regulation, and data is freely available due to a retrospective analysis from the MIMIC-III. The MIMIC-III database has approved the permission, as shown in https://physionet.org/content/mimiciii/1.4/.

### Variable extraction

The raw data on the ICU patients were obtained by using pgAdmin Postgre SQL tools. Then, Stata software (13.0) was used for data cleaning and pretreatment. The following data on clinical characteristics were extracted: age, sex, race, days of ICU stay, insurance, ICU type, systolic and diastolic BP, type of cerebral hemorrhage, traumatic hemorrhage, comorbidities and blood parameters. Then, blood biochemical indices, including glucose, partial thromboplastin time (PTT), prothrombin time (PT), international normalized ratio (INR), red blood cell (RBC) count, white blood cell (WBC) count and platelet counts, were included after ICU admission. In the present analysis, we used the maximum and minimum values for describing all continuous variables due to the high sampling frequency.

For categorical variables, ICU types included trauma/surgical intensive care unit (TSICU), insurance intensive care unit (MICU), surgical intensive care unit (SICU), cardiovascular surgery rehabilitation unit (CSRU) and cardiology care unit (CCU); traumatic hemorrhage was defined as “yes” or “no”; type of cerebral hemorrhage mainly included “extradural hemorrhage”, “intracranial hemorrhage”, “subdural hemorrhage”, “subarachnoid hemorrhage” and “multiple hemorrhage”; comorbidities were obtained, including hypertension, heart failure, myocardial infarction, malignancy, cerebral infarction, diabetes and chronic kidney disease.

### The outcomes of follow-up

To assess the risk of short-term and long-term mortality in patients with cerebral hemorrhage, 1-month mortality and 1-year mortality were defined as the endpoints. After these patients recovered from treatment and were discharged from the hospital, their survival status was recorded. The follow-up methods are described in the MIMIC-III database.

### Statistical analysis

First, all included patients were classified into two groups based on whether they were diagnosed with “hypertension”. All variables in our study were compared between the two groups (patients with hypertension *vs.* without hypertension). Normally and non-normally distributed continuous variables were summarized as the mean ± SD and the median (interquantile range), respectively.

Then, we constructed a smooth curve for associations between blood BP levels and risks of 1-month and 1-year mortality. Furthermore, we performed three multivariate logistic regression models to evaluate the associations between BP and the risks of 1-month mortality and 1-year mortality. In Model 1, age and sex were adjusted. In Model 2, age, sex, race and insurance were adjusted. In Model 2, age, sex, race, insurance, heart failure, myocardial infarct, malignant, cerebral infarction, diabetes, and chronic kidney disease were adjusted. Finally, in the stratification analysis, we used “type of cerebral hemorrhage” as a stratification variable for evaluating these associations between BP and mortality risks. Stata 13.0 and EmpowerStats 3.0. were used for all statistical analyses in our study. A *P* value < 0.05 was considered to be statistically significant.

## Results

### Clinical characteristics

A total of 1085 patients with cerebral hemorrhage were enrolled in our study, as shown in the study flow diagram (Fig. 1). Among these patients, the median age was 65.58 years, and 609 (56.13%) were female. These patients were further classified into the group with hypertension (N = 531) and the group without hypertension (N = 554), as shown in Table 1. The patients with hypertension were likely to be older, Asian and Black and had worse health insurance and higher systolic BP-max than the group without hypertension. However, there were no significant differences in 1-month mortality or 1-year mortality between the two groups.

**Table 1:** Clinical characteristics

Variable	All	NO hypertension	Hypertension	*P* value
	*N* = 1085	*N* = 554	*N* = 531	
Age (year)	65.58 (51.75–78.05)	59.49 (45.06–75.84)	72.04 (59.54–79.38)	< 0.001
Sex (male), n (%)	609 (56.13%)	323 (58.30%)	286 (53.86%)	0.140
Systolic BP-max (mmHg)	181.00 (162.00–201.00)	175.00 (157.00–197.00)	185.00 (167.00–204.50)	< 0.001
Systolic BP-min (mmHg)	84.50 (0.00–99.00)	82.50 (0.00–97.00)	86.00 (0.00–99.00)	0.315
Diastolic BP-max (mmHg)	97.00 (84.00–117.00)	96.00 (82.25–115.00)	99.00 (85.00–119.50)	0.089
Diastolic BP-min (mmHg)	40.00 (0.00–48.00)	40.00 (0.00–49.00)	40.00 (0.00–48.00)	0.291
**Race**				0.017
Asian, n (%)	26 (2.40%)	7 (1.26%)	19 (3.58%)	
Black, n (%)	61 (5.62%)	24 (4.33%)	37 (6.97%)	
White, n (%)	758 (69.86%)	395 (71.30%)	363 (68.36%)	
Other, n (%)	240 (22.12%)	128 (23.10%)	112 (21.09%)	
**Insurance**				< 0.001
Government, n (%)	35 (3.23%)	24 (4.33%)	11 (2.07%)	
Medicaid, n (%)	92 (8.48%)	46 (8.30%)	46 (8.66%)	
Medicare, n (%)	513 (47.28%)	209 (37.73%)	304 (57.25%)	
Private, n (%)	415 (38.25%)	251 (45.31%)	164 (30.89%)	
Self Pay, n (%)	30 (2.76%)	24 (4.33%)	6 (1.13%)	
**Firts Careunit**				< 0.001
CCU, n (%)	30 (2.76%)	11 (1.99%)	19 (3.58%)	
CSRU, n (%)	70 (6.45%)	46 (8.30%)	24 (4.52%)	
MICU, n (%)	175 (16.13%)	87 (15.70%)	88 (16.57%)	
SICU, n (%)	470 (43.32%)	202 (36.46%)	268 (50.47%)	
TSICU, n (%)	340 (31.34%)	208 (37.55%)	132 (24.86%)	
**Traumatic hemorrhage**				< 0.001
NO trauma, n (%)	698 (64.33%)	310 (55.96%)	388 (73.07%)	
Trauma, n (%)	387 (35.67%)	244 (44.04%)	143 (26.93%)	
**Type of cerebral hemorrhage**				< 0.001
Extradural hemorrhage, n (%)	3 (0.28%)	1 (0.18%)	2 (0.38%)	
Intracranial hemorrhage, n (%)	475 (43.78%)	210 (37.91%)	265 (49.91%)	
Subdural hemorrhage, n (%)	230 (21.20%)	121 (21.84%)	109 (20.53%)	
Subarachnoid hemorrhage, n (%)	341 (31.43%)	191 (34.48%)	150 (28.25%)	
Multiple hemorrhage, n (%)	36 (3.32%)	31 (5.60%)	5 (0.94%)	
**Comorbidities**				
Heart failure, n (%)	139 (12.81%)	70 (12.64%)	69 (12.99%)	0.860
Myocardial infarct, n (%)	18 (1.66%)	14 (2.53%)	4 (0.75%)	0.022
Malignant, n (%)	36 (3.32%)	26 (4.69%)	10 (1.88%)	0.010
Cerebral infarction, n (%)	57 (5.25%)	25 (4.51%)	32 (6.03%)	0.264
Diabetes, n (%)	10 (0.92%)	6 (1.08%)	4 (0.75%)	0.570
Chronic kidney disease, n (%)	11 (1.01%)	9 (1.62%)	2 (0.38%)	0.040
**Blood parameters**				
Glucose-max (mg/dL)	178.00 (147.00–219.00)	175.00 (145.00–216.00)	182.00 (151.50–225.50)	0.011
Glucose-min (mg/dL)	93.00 (81.00–107.00)	92.00 (80.00–104.00)	95.00 (83.00–110.00)	0.001
PTT-max (sec)	30.20 (26.80–36.90)	30.95 (27.20–39.80)	29.60 (26.70–34.60)	< 0.001
PTT-min (sec)	24.60 (22.70–26.70)	24.55 (22.60–26.80)	24.60 (22.80–26.55)	0.730
PT-max (sec)	13.90 (13.20–15.40)	14.10 (13.30–15.60)	13.80 (13.10–15.00)	0.003
PT-min (sec)	12.60 (12.10–13.10)	12.50 (12.10–13.00)	12.60 (12.10–13.10)	0.338
INR PT-max	1.30 (1.20–1.50)	1.30 (1.20–1.50)	1.20 (1.10–1.50)	< 0.001
INR PT-min	1.10 (1.00–1.10)	1.10 (1.00–1.10)	1.10 (1.00–1.10)	0.427
RBC-max (m/uL)	4.10 (3.74–4.45)	4.09 (3.73–4.43)	4.12 (3.76–4.48)	0.408
RBC-min (m/uL)	3.16 (2.80–3.64)	3.09 (2.70–3.57)	3.24 (2.89–3.68)	< 0.001
Platelet count-max (K/uL)	313.00 (230.00–449.00)	311.00 (226.00–461.75)	313.00 (230.50–440.00)	0.974
Platelet count-min (K/uL)	172.00 (122.00–218.00)	162.00 (109.25–211.00)	180.00 (134.00–224.00)	< 0.001
WBC-max (K/uL)	14.90 (11.90–19.00)	15.55 (12.22–19.97)	14.30 (11.55–18.10)	< 0.001
WBC-min (K/uL)	7.70 (5.90–9.90)	7.60 (5.80–9.60)	7.90 (6.20–10.20)	0.078
**Mortality**				
1-month mortality, n (%)	324 (29.86%)	166 (29.96%)	158 (29.76%)	0.940
1-year mortality, n (%)	413 (38.06%)	214 (38.63%)	199 (37.48%)	0.696

*BP* blood pressure, *ICU* Intensive Care Unit, *CCU* Cardiology Care Unit, *CSRU* Cardiovascular Surgery Rehabilitation Unit, *MICU* Medical Intensive Care Unit, *SICU* Surgical Intensive Care Unit, *TSICU* Trauma/Surgical Intensive Care Unit, *PTT* partial thromboplastin time, *PT* prothrombin time, *INR* international normalized ratio, *RBC* Red blood cell count, *WBC* White blood cell count

### Systolic and diastolic BP-min contributed to a reduced risk of 1-month morality and 1-year mortality

The logistic regression analysis revealed significantly inverse relationships between systolic BP-min (adjusted odds ratio (OR) = 0.986, 95% CI 0.983–0.989, P < 0.001; Model 3) and diastolic BP-min (OR = 0.975, 95% CI 0.968–0.981, P < 0.001; Model 3) and the risk of 1-month mortality as shown in Table 2, after adjusting for age, sex, race, insurance, heart failure, myocardial infarct, malignancy, cerebral infarction, diabetes and chronic kidney disease. Systolic and diastolic BP-max were not associated with the risk of 1-month mortality. Consistently, associations of systolic BP-min (OR = 0.987, 95% CI 0.983–0.990, P < 0.001; Model 3) and diastolic BP-min (OR = 0.975, 95% CI 0.968–0.981, P < 0.001; Model 3) with the risk of 1-year mortality also exist, as shown in Table 3.

**Table 2:** Logistic regression analysis for the association of BP with risk of 1-month mortality

Variables	NO adjusted		Model 1		Model 2		Model 3	
	OR (95%CI)	*P* value	OR (95%CI)	*P* value	OR (95%CI)	*P* value	OR (95%CI)	*P* value
Systolic BP-max	1.000 (0.996, 1.004)	0.959	0.999 (0.995, 1.003)	0.727	1.001 (0.996, 1.005)	0.753	1.001 (0.997, 1.006)	0.625
Systolic BP-min	0.987 (0.984, 0.990)	< 0.001	0.987 (0.984, 0.990)	< 0.001	0.986 (0.983, 0.989)	< 0.001	0.986 (0.983, 0.989)	< 0.001
Diastolic BP-max	0.994 (0.989, 0.998)	0.006	0.995 (0.991, 1.000)	0.030	0.996 (0.991, 1.000)	0.056	0.996 (0.991, 1.000)	0.073
Diastolic BP-min	0.976 (0.970, 0.982)	< 0.001	0.977 (0.971, 0.983)	< 0.001	0.975 (0.969, 0.982)	< 0.001	0.975 (0.968, 0.981)	< 0.001

Model 1: adjust for age and sex

Model 2: adjust for age, sex, race and insurance

Model 3: adjust for age, sex, race, insurance, heart failure, myocardial infarct, malignant, cerebral infarction, diabetes and chronic kidney disease

*BP* blood pressure

**Table 3:** Logistic regression analysis for the association of BP with risk of 1-year mortality

Variables	NO adjusted		Model 1		Model 2		Model 3	
	OR (95%CI)	*P* value	OR (95%CI)	*P* value	OR (95%CI)	*P* value	OR (95%CI)	*P* value
Systolic BP-max	1.002 (0.998, 1.005)	0.424	1.001 (0.996, 1.005)	0.801	1.002 (0.997, 1.006)	0.446	1.002 (0.998, 1.006)	0.403
Systolic BP-min	0.988 (0.985, 0.991)	< 0.001	0.988 (0.985, 0.991)	< 0.001	0.987 (0.984, 0.990)	< 0.001	0.987 (0.983, 0.990)	< 0.001
Diastolic BP-max	0.998 (0.994, 1.002)	0.240	1.000 (0.996, 1.004)	0.824	1.000 (0.996, 1.004)	0.996	1.000 (0.996, 1.004)	0.929
Diastolic BP-min	0.976 (0.970, 0.982)	< 0.001	0.977 (0.971, 0.983)	< 0.001	0.976 (0.969, 0.982)	< 0.001	0.975 (0.968, 0.981)	< 0.001

Model 1: adjust for age and sex

Model 2: adjust for age, sex, race and insurance

Model 3: adjust for age, sex, race, insurance, heart failure, myocardial infarct, malignant, cerebral infarction, diabetes and chronic kidney disease

*BP* blood pressure

We used stratified analysis to evaluate further the effect of “type of hemorrhage” on these associations. As shown in Tables 4–5, we observed that in patients with “intracranial hemorrhage”, “subdural hemorrhage” and “subarachnoid hemorrhage”, higher systolic and diastolic BP-min were still associated with a reduced risk of 1-month mortality and 1-year mortality. Similarly, systolic and diastolic BP-max were almost entirely unrelated to the two morality risks.

**Table 4:** Subgroup analysis for the association of BP with risk of 1-month mortality by using “type of hemorrhage” as the covariat

Variables	Intracranial (*n* = 475)		Subdural (*n* = 230)		Subarachnoid (*n* = 341)	
	OR (95%CI)	*P* value	OR (95%CI)	*P* value	OR (95%CI)	*P* value
Systolic BP-max	0.996 (0.989, 1.002)	0.204	1.017 (1.004, 1.030)	0.009	1.000 (0.992, 1.009)	0.917
Systolic BP-min	0.986 (0.981, 0.991)	< 0.001	0.981 (0.973, 0.989)	< 0.001	0.989 (0.983, 0.995)	< 0.001
Diastolic BP-max	0.986 (0.979, 0.994)	< 0.001	1.009 (0.997, 1.020)	0.131	1.001 (0.993, 1.009)	0.892
Diastolic BP-min	0.974 (0.964, 0.984)	< 0.001	0.963 (0.946, 0.980)	< 0.001	0.982 (0.970, 0.993)	0.002

Adjust for age, sex, race, insurance, heart failure, myocardial infarct, malignant, cerebral infarction, diabetes, and chronic kidney disease

*BP* blood pressure

**Table 5:** Subgroup analysis for the association of BP with risk of 1-year mortality by using “type of hemorrhage” as the covariat

Variables	Intracranial (*n* = 475)		Subdural (*n* = 230)		Subarachnoid (*n* = 341)	
	OR (95%CI)	*P* value	OR (95%CI)	*P* value	OR (95%CI)	*P* value
Systolic BP-max	0.999 (0.993, 1.006)	0.803	1.020 (1.007, 1.032)	0.001	0.999 (0.991, 1.007)	0.860
Systolic BP-min	0.985 (0.980, 0.990)	< 0.001	0.979 (0.971, 0.988)	< 0.001	0.990 (0.984, 0.996)	< 0.001
Diastolic BP-max	0.994 (0.988, 1.000)	0.066	1.018 (1.005, 1.030)	0.005	1.001 (0.993, 1.009)	0.809
Diastolic BP-min	0.972 (0.962, 0.982)	< 0.001	0.953 (0.935, 0.971)	< 0.001	0.983 (0.972, 0.995)	0.004

Adjust for age, sex, race, insurance, heart failure, myocardial infarct, malignant, cerebral infarction, diabetes, and chronic kidney disease

*BP* blood pressure

### The smooth curve suggested an approximate L-shaped relationship between systolic and diastolic BP-min and mortality risk.

**Fig. 2:**
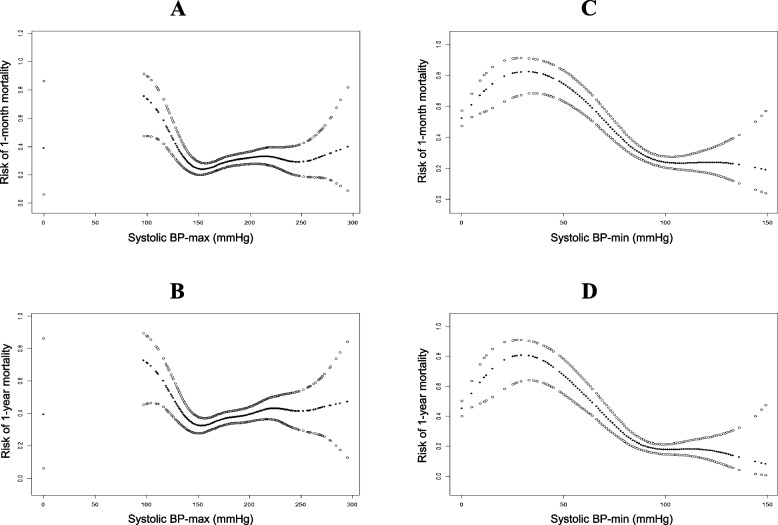
**A** Smooth curve for association between systolic BP-max (mmHg) and risk of 1-month mortality in ICU patients with cerebral hemorrhage. **B** Smooth curve for association between systolic BP-max (mmHg) and risk of 1-year mortality in ICU patients with cerebral hemorrhage. **C** Smooth curve for association between systolic BP-min (mmHg) and risk of 1-month mortality in ICU patients with cerebral hemorrhage. **D** Smooth curve for association between systolic BP-min (mmHg) and risk of 1-year mortality in ICU patients with cerebral hemorrhage

**Fig. 3:**
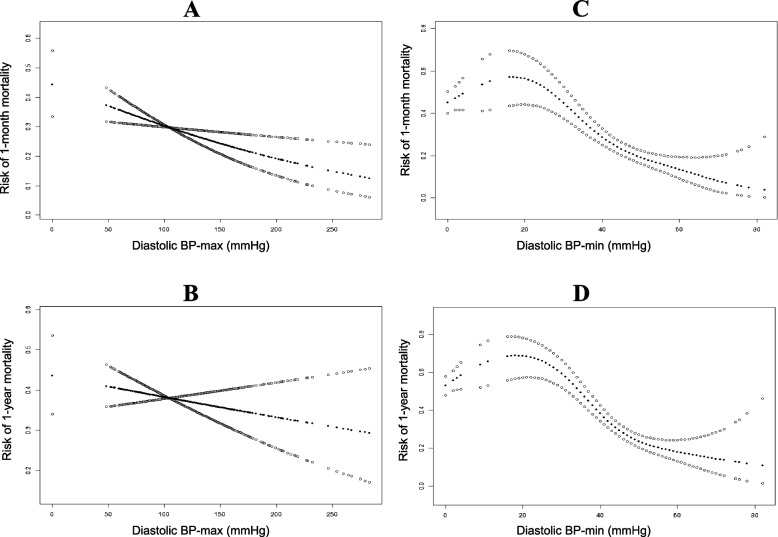
**A** Smooth curve for association between diastolic BP-max (mmHg) and risk of 1-month mortality in ICU patients with cerebral hemorrhage. **B** Smooth curve for association between diastolic BP-max (mmHg) and risk of 1-year mortality in ICU patients with cerebral hemorrhage. **C** Smooth curve for association between diastolic BP-min (mmHg) and risk of 1-month mortality in ICU patients with cerebral hemorrhage. **D** Smooth curve for association between diastolic BP-min (mmHg) and risk of 1-year mortality in ICU patients with cerebral hemorrhage

To further investigate the association between BP and mortality risk in these ICU patients, smooth curve analysis was used and it suggested an approximate L-shaped association of systolic BP with the risk of 1-month mortality and 1-year mortality (Fig. 2 A-D). Similar results existed for the associations of diastolic BP-min (Fig. 3 C, D), rather than diastolic BP-max (Fig. 3 A, B), with the risk of 1-month mortality and 1-year mortality. We found that the systolic BP-min at the lowest risk of death was 100 mmHg. When systolic BP-min was less than 100 mmHg, increased systolic BP-min significantly contributed to lower risks of 1-year mortality and 1-year mortality. However, the mortality risks did not decrease or decreased very little if the systolic BP-min value was more than 100 mmHg. Diastolic BP-min had an almost gradual downward trend for 1-month mortality and 1-year mortality.

In addition, we observed that the systolic BP-max at the lowest mortality risk was 150 mmHg. When systolic BP-max was less than 150 mmHg, increased systolic BP-max was significantly associated with reduced risks of 1-year mortality and 1-year mortality, whereas the mortality risks were significantly elevated if the systolic BP-max value was greater than 150 mmHg. These results might suggest that reducing systolic BP in the range of 100–150 mmHg may have a lower risk of short-term and long-term death in patients with cerebral hemorrhage.

## Discussion

A long-term concern among clinicians is that early and rapidly lowering BP might cause ischemic damage to the brain after acute stroke. However, a clinical trial from the main phase Intensive Blood Pressure Reduction in Acute Cerebral Hemorrhage Trial (INTERACT2) suggested that rapidly lowering BP < 140 mmHg had a significant effect on functional recovery for ICH patients with hypertension (SBP, 150–220 mmHg) [22]. Furthermore, some other studies have also shown good safety of a rapid BP reduction in ICH patients, such as the Stroke Acute Management With Urgent Risk-Factor Assessment and Improvement-Intracerebral Hemorrhage Study (SAMURAI-ICH Study) and the Intracerebral Hemorrhage Acutely Decreasing Arterial Pressure (ICH-ADAPT) trial [23, 24]. Moreover, there is evidence supporting that greater systolic BP variability within the first 24 h after admission was independently associated with the worse outcomes in ICH patients and suggested a need to monitor and control BP fluctuations in the routine clinical care of ICH patients [25–27]. Inconsistently, in the retrospective analysis of the MIMIC-III database, our logistic regression analysis demonstrated that elevated systolic and diastolic BP during the acute phase of ICH in ICU patients are related to lower risks of 1-month mortality and 1-year mortality and that controlling systolic BP values within 100–150 mmHg was associated with the lowest risk of death. These results suggested a potential beneficial effect of controlling systolic BP in a stable range (100–150 mmHg) for ICH patients in the ICU. Lowering systolic BP rapidly may be not conducive to a good short-term and long-term prognosis for ICU patients with cerebral hemorrhage.

Interestingly, some previous studies have also pointed out that rapidly lowering BP might cause adverse outcomes in patients with acute ICH. For instance, a prospective cohort study suggested that a rapid decline in mean BP within 24 h contributed to an increased risk of hospital mortality in 105 ICH patients [16]. Another study demonstrated that an early reduction of systolic BP was not related to the death risk at 3 weeks in a cohort of 688 patients with ICH [28]. Consistently, our results showed that there was a significantly lower risk for one-month death after systolic BP reduction, especially when the systolic BP was in the range of 100–150 mmHg. These previous inconsistent conclusions may be related to differences in population selection, sample size, study design, concomitant diseases and BP measurement methods, especially differences in the time of measuring BP from stroke onset. Our study samples were collected from the ICU, and these patients may have had other chronic and serious diseases. The BP value was measured from the ECG monitor, and we only collected the lowest and highest values during the ICU stay for analysis. From the perspective of the mechanism, the potential beneficial effects of lowering BP in ICH patients are to reduce the hydrostatic pressure driving the hemorrhage and thus reduce its expansion. BP reduction also lowers the risks of rebleeding, perihaematoma edema and early stroke recurrence [29–31]. Our results supported the beneficial effect of slowly lowering BP in the range of 100–150 mmHg on the prognosis of patients with cerebral hemorrhage. In fact, the internationally recognized BP control standard after ICH is below 140/90 mmHg [32]. Especially for patients with hypertensive intracerebral hemorrhage. Many clinicians worried that too-low BP would cause symptoms of cerebral ischemia and cerebral infarction if the BP dropped too low. Therefore, they set this standard at about systolic BP at 140–160 mmHg for acute ischemic stroke [33, 34]. Our research evidence further supported the conclusion that maintaining BP at a relatively normal level is beneficial to the long-term and short-term mortality risk in ICH patients.

A notable strength of our study was the large-sample data from the MICMIC-III database from a large number of ICU patients with cerebral hemorrhage, which ensures reliability and precision for the association of BP with mortality risk. This retrospective cohort study estimated the real-world prognosis of these patients with cerebral hemorrhage and observed an approximate L-shaped relationship between systolic BP levels and short-term and long-term mortality risks in these ICU patients. Another strength is that our study samples were from different races in the United States, which can increase the generalizability of our results. Furthermore, confounding factors for mortality risk, such a medical insurance, concomitant cardiovascular disease (heart failure, myocardial infarction, cerebral infarction), diabetes, malignancy and chronic kidney disease, were controlled, which further ensured the reliability of our results.

Our study, however, also had several limitations. Firstly, due to the inherent shortcomings of retrospective studies, selection bias might be caused by the fact that approximately 2/3 of patients with cerebral hemorrhage had missing key variables and were excluded from the study cohort (Fig. 1). Secondly, we did not collect BP values in the early stage after cerebral hemorrhage, which is the stage most closely related to the prognosis of patients. Only the maximum and minimum BP values during the ICU stay were included due to the high sampling frequency of the BP record. Thirdly, the endpoint of this study was only all-cause mortality, and disease-specific mortality was not analyzed. Fourthly, these included patients in MIMIC-III database are mainly composed of Americans that the Asian and Black samples are relatively small, which makes it impossible to fully extrapolate our results to other race groups.

## Conclusion

This study demonstrated that reducing BP has protective effects against 1-month and 1-year mortality risks after cerebral hemorrhage in ICU patients. Controlling the systolic BP level at 100–150 mmHg has the optimum protective effect on reducing short-term and long-term mortality.

## Data Availability

The data used in the present study are publicly available through the MIMIC-III database: https://mimic.mit.edu/docs/iii/.
